# Great Green Transition and Finance

**DOI:** 10.1007/s10272-020-0896-y

**Published:** 2020-06-07

**Authors:** Claudia Kemfert, Dorothea Schäfer, Willi Semmler

**Affiliations:** 1 Depart. Energy, Traffic and Environment, German Institute for Economic Research DIW Berlin, Mohrenstraße 58, 10117 Berlin, Germany; 2 German Institute for Economic Research DIW Berlin, Mohrenstraße 58, 10117 Berlin, Germany; 3 Depart. of Economics Graduate Faculty, New School for Social Research, New School 65 Fifth Ave, New York, NY 10003 USA

## Abstract

European governments are struggling to regain economic strength in the coronavirus pandemic as in many countries the number of new infections seems to gradually subside. Growth rates deep in the red call for a reconstruction programme when the crisis is finally manageable and economic activity can resume. Amidst this, there are again influential groups that claim “this is not the time to insist on strict climate protection goals”. On the contrary, the ongoing COVID-19 crisis has clearly illustrated what climate disasters, often occurring locally, could do to the life of citizens. The reconstruction programme needs to initiate the great green transition. The transformation from a climate-distorting to a climate-protecting economy opens up investment opportunities and points to financing needs comparable with those necessary for the rebuilding of the European economy after World War II. The great green transition is a unique chance to pursue policies for a new and sustainable growth regime.

European governments are striving to meet the ambitious goals of the Paris Climate Agreement of 2015. The German government wants to limit $CO_2$ emissions so that the global temperature increase does not exceed 1.5 degrees Celsius. How German society can achieve this goal is still an open question. However, concrete steps must be decided upon urgently, and rapid implementation is key.

Within this scenario, the financial sector faces a dilemma. The climate crisis is a source of financial market instability. Without strict greenhouse gas emission regulation, climate disruption risks increase that are potentially depreciating the assets of banks and other financial institutions. However, with stricter regulation and higher $CO_2$ emission pricing, there is a risk that the legacy investments of financial institutions in the fossil fuel sectors will be devaluated, a risk that increases with stricter regulation and rising $CO_2$ emission pricing.

**Claudia Kemfert**, German Institute for Economic Research (DIW Berlin); and German Advisory Council on the Environment (SRU), Berlin, Germany.

**Dorothea Schäfer**, German Institute for Economic Research (DIW Berlin), Germany; Jönköping International Business School, Sweden.

**Willi Semmler**, New School for Social Research, New York, USA; University of Bielefeld, Germany.

On the other hand, the transformation from a climate-distorting into a climate-protecting economy opens up new investment opportunities. The resulting financing needs will be in a range that is likely to be comparable only with the volumes needed for rebuilding the German economy after the World War II, or for the reconstruction of the East German economy after the reunification. The transformation requires investments in all areas of the economy: low-carbon and climate resilient infrastructure, including energy, transport, drinking water, sanitation and telecommunication; human capital investments (for planning and implementation, advice, risk management, auditing and regulatory capacity); refurbishment and redesign of public and private buildings (transformation of the construction industry); agriculture; tourism and many other areas (Dullien, 2019a; Krebs, 2020). The investment requirements are so huge that it is justified to call the path to a carbon-free and sustainable economy the 'great green transition'. Eventually, during the transition period, the German corporate sector, which joined the German state and private households in being a net-saving sector, may again become a net-investing sector (Flassbeck, 2019).

Along with the huge opportunities embedded in the financing of the great green transition come huge uncertainties for financial institutions, however. To a large extent, the transition path is unknown. Success in transforming the economy will require, and be highly dependent on, innovative firms inventing, producing and selling climate-protecting technologies and products. Accordingly, financial institutions face huge innovation risks when funding the great green transition. Innovative companies are more financially constrained than those firms using and selling proven technologies and products (Jensen et al., 2019; Schäfer et al., 2017). The reason for severe funding gaps is the huge uncertainty revolving around the question of long-term sustainability and market success of innovative products and technologies. Therefore, banks and institutional investors funding climate tech firms face two kinds of depreciation risks. At the one end, if the financial industry conducts a wait-and-see strategy and sticks to their traditional investment policy, stranded legacy assets may become a huge source of future losses should loans and other funding of traditional fossil fuel investments become non-performing. At the other end, if the financial industry immediately switches to funding only innovative, yet not financially self-sustainable, climate tech firms with new technologies, it faces the typical first-mover risk in completely new, innovative and unknown areas of business.

Thus, the crucial question is: how can the necessary investments be initiated, carried out and funded without generating a situation in which devaluation and depreciation endangers the stability of the financial sector? In 2019, the German climate cabinet proposed a mix of policies – innovation support for green energy, fiscal policies (tax and subsidies), carbon pricing and issuance of green bonds. Often, German climate policy is considered ‘too little and too late’, yet it seems to be an important first step in the right direction as it focuses on a mix of instruments instead of just one. Indeed, a mix of policies is required to achieve the great green transition. Redirecting innovation policy and innovation finance, large scale issuance of green bonds, active fiscal policy, supportive monetary and credit policies, fair transitions of employees in old industries in the labour market and better insurance policies are necessary to allow a faster transition to a low-carbon economy.

## Macro instruments for the great green transition

### Carbon pricing and green bonds

Sovereign institutions and their chosen policy instruments will play a decisive role in mitigating the multiple dilemmas arising around a timely and sufficient provision of funds for the great green transition. Why is it so important to use a mix of policy instruments at the same time, for example, $CO_2$ pricing and green investments? The pricing of $CO_2$ emissions is now largely undisputed. Disputed, however, is how the pricing should be organised. Some advocates favour focusing only on the trading of certificates and object to the introduction of a $CO_2$ tax.

However, emissions trading poses severe problems: the prices of the certificates on the financial markets are subject to extreme fluctuations, so they are very volatile. Studies with existing data suggest that certificate prices are many times more volatile than stock prices. The stock markets exhibit anything but low volatility. Large fluctuations in prices mean high planning uncertainties for investments in the great green transition (Kemfert et al., 2019). Moreover, and paradoxically, if the price cycle of certificates is at the lower end and the $CO_2$ price is very low, as has happened in the past, this would incentivise firms to slow down, if not abolish, their efforts to increase green investments for achieving lower $CO_2$ emissions (Dullien, 2020).

By contrast, the effect of the $CO_2$ tax is steadier and more stable, thus complementing and supporting green investments. The tax can be implemented quickly, without an extended preparation phase. Moreover, as the private sector can limit the tax burden by reducing $CO_2$ emissions, the tax is incentive compatible and does not suffer from the risk of reversing incentives.

Taken alone, $CO_2$ taxes are not effective enough to facilitate the necessary breakthrough for sustainability policy. In fact, the tax must be very high to be effective. The required level is not US$20-30 per tonne of $CO_2$ (or about US$50 as it is now planned for Germany in 2025) but rather US$80-100, as calculated by Heal and Schlenker (2019). At lower levels, the $CO_2$ tax would only very slowly initiate the needed substitution processes. In addition, firms may pass on the tax via price markups. Instead of adjusting their own production processes towards lower $CO_2$ emissions, companies with enough market power could easily increase prices for their customers.

In tandem with green bond issues at a large scale and used for public green investment, the revenues of a carbon tax can have side benefits that are highly welcome, even if they do not initiate changes in behaviour. The revenues could be used to reduce other taxes that are incompatible with sustainability or to correct unwanted distributional effects of $CO_2$ pricing. As higher fossil fuel prices affect poorer citizens disproportionally, tax revenues could serve to compensate them. In addition, the revenues could create an allocative effect if they are paid out as a citizen dividend, i.e. a per capita refund of revenue. For citizens who themselves cause only limited $CO_2$ emissions, the balance between the $CO_2$ tax paid and the citizen dividend would be positive. Not only does this create a strong incentive to reduce $CO_2$ emissions, it also facilitates a fairer transition to a less carbon intensive economy.

*A sustainability policy needs green bonds*

Public ‘green investments’ financed by ‘green government bonds’, i.e. bonds issued by public agencies on behalf of sovereign institutions, are an important complementary tool to $CO_2$ pricing. The Federal Ministry of Finance in Germany recently pushed forward considerations in this direction. Green bonds are also an essential part of the Green Finance policy of the EU Commission (Claringbould et al., 2019). The Action Plan aims to develop an EU Green Bond Standard, as well as benchmarks for low-carbon investment strategies and climate-related reporting.

In designing the policy towards decarbonisation, the relationship between $CO_2$ pricing and green investments, as well as the potential value-added of their simultaneous use, is often ignored. However, some governments have long recognised the importance and effectiveness of a simultaneous use in their economic policy for sustainability. Further, the benefits of a joint use of $CO_2$ pricing and green investments are starting to become common knowledge in scientific research. For example, Heine et al. (2019) argue that public green bonds are more effective when combined with a carbon tax.

*Fiscal space should be used for countercyclical green investments*

Furthermore, it is currently a good time to issue new public debt. Countries like Germany – and other Northern EU countries – have enough fiscal space to pursue countercyclical green investments, even when fighting the coronavirus crisis causes extraordinary large expenses. Other countries, especially those with emerging economies, lack such freedom. According to studies by the World Bank and the International Monetary Fund (IMF), the comparison of long-term growth and the interest rates of a country provides a benchmark, or an assessment of whether or not there is room for fiscal expansion. If the interest rate is lower than the growth rate, there is sufficient leeway. In this case, no long-run sovereign debt problem will arise when issuing green bonds for co-funding the great green transition.

In view of the expected economic downturn and low or negative interest rates, Germany could and should increase public investment. The World Bank, the IMF, the European Central Bank (ECB) and the Organisation for Economic Co-operation and Development are also calling for higher public investments from Germany in order to counteract the predicted global economic downturn and for reducing Germany's export dependency. Above all, the favourable financing environment should be used to issue bonds for funding green investments.

The issuance of green government bonds is also a good strategy for Germany in the medium term. From an international perspective, Germany has extremely low capital costs due to low, if not negative, interest rates. Germany could have a comparative cost advantage in the production of technologies for the transformation of the energy system. Low-cost trade credits to other countries, given the low domestic cost of capital, could also help unlock new demand for climate-friendly export goods. Therefore, in sum, using a combination of $CO_2$ tax and green public bonds to initiate the urgently needed boost for the great green transition seems to be a reasonable strategy in fighting the climate change and in overcoming the economic weakness from the coronavirus pandemic.

*Fair intergenerational burden sharing*

Tax increases, especially in sufficient amounts, are politically difficult to enforce if the benefits to those affected are not directly visible. Scientific research discusses whether a sufficiently high $CO_2$ tax would cause unequal burdens not just within the current generation, but also between present and future generations. Intergenerational fairness implies that it is not only the present generation, but also future generations, that benefit from state spending on education, all kinds of infrastructure, energy supply, digitisation and other investments with long-term beneficial effects. For that reason, future beneficiaries should also participate in the financing of climate-protecting investments. The current financial market conditions, with extremely low, if not negative, interest rates, ensure that the future generations' repayment burden is low. Even very long-term government bonds have negative interest rates at present. If the growth rate is higher than the interest rate, the debt ratio, i.e. the ratio of public debt to gross domestic product, decreases. With negative interest rates, future generations will have to repay less than the funds that were originally made available to the state when it took on the debt.

Consequently, scientific research must not only focus on the intergenerational costs, when discussing the issuance of public debt, but also consider the intergenerational benefits. Since a $CO_2$ tax has only limited effectiveness and does not solve the intergenerational burden sharing problem, green bonds are a crucially important instrument of sustainability policy.

*Use of the revenue from green bonds*

Of course, money itself is not 'green'. What makes money green is its investment in projects that contribute to the great green transition. Thus, if governments issue green public bonds, they implicitly commit to use the revenues from those bonds for green investments. The question arises as to which green investments the bond revenues should be allocated:

- Governments could allocate the revenues to development banks, like the European Investment Bank. These financial institutions could then issue government-sponsored bonds for which the state guarantees all, or parts of, debt service and repayment. This implies lower risk premiums for those bonds than for privately issued bonds. As a result, investments into new energy supply become more feasible.

- Larger companies mainly finance themselves through the issuance of shares or bonds, but small and medium-sized enterprises (SMEs) in Europe rely strongly on bank loans. Loan guarantee programmes are an instrument to reduce interest rates and could be funded by green bond revenues. In other words, the revenues would be used to cover the default risk of loans, given the loan is used for green investment purposes. This would ensure that smaller, often credit-constrained, innovative companies operating in the new manufacturing sectors receive more favourable funding terms (see e.g. Jensen et al., 2019).

- Revenues could go directly into climate-protecting public infrastructure. This implies that the state would build, own, maintain and manage the infrastructure, e.g. the grids for renewable energy distribution.

- Revenues could be used to set up, subsidise and regulate public-private utility companies generating green energy, such as a solar energy utility company. For example, Tesla's planned new factory in Brandenburg could be required to use 100% renewable energy instead of gas power plants. With rising $CO_2$ prices and concrete support programmes, solar process heat is profitable today at €0.05 per kWh. Another possibility is the combination of photovoltaic and a high temperature heat pump or a hybrid collector, e.g. for drying, painting, cooling and heating. Alternatively, biomass (boiler), biogas (CHP) or deep geothermal energy could be used for power and heat production.

- Direct subsidies for small-scale innovative firms introducing new types of green energy.

- Large investments are necessary in education, e.g. setting up educational tracks that provide the appropriate skills for the transition, such as green engineers, green business managers and green rating specialists.

- Governments need to invest in precautionary protective measures against disasters (extreme weather events such as heat waves, forest fires, flooding, storms and other disasters), with the issuance of green bonds providing the means.

### Greening central bank policy

In the current low inflationary environment, the ECB could become – and such a strategy appears to be emerging under Christine Lagarde – an active player in developing the market for green government bonds. There are various ways to proceed. For example, De Grauwe (2019) recommends that the ECB should replace old bonds within its quantitative easing programme with new 'environmental bonds' that are specifically issued to fund environmental projects. As the ECB would only substitute environmental bonds for old bonds, there would be no new money created, and inflation would not be affected.

Fratzscher et al. (2017) and McKibbin et al. (2017) elaborate on the importance of monetary policy for climate policy in general. Many monetary policy observers argue that inflation targeting is an appropriate goal, even if a climate disaster has hit the economy. Yet, climate disasters on account of extreme weather events and the subsequent recovery efforts often cause severe shortages and thus higher inflation rates. Due to the fact that the problem is on the supply side, central banks are required, as it is naturally part of their mandate to facilitate credit flows and provide credit with low interest rates. Against this backdrop, inflation targeting seems to be of secondary importance when the economy is struggling with negative shocks on the supply side. This ranking holds for the current pandemic crisis and equally so for the climate crisis.

When climate disasters occur, households and firms usually face severe credit constraints. Moreover, collateral value declines due to value losses in housing and real estate. Thus, it is hard to obtain credit, or only at a very large risk premium. Developing precautionary tools to overcome such constraints should be a major task of central banks.

Monetary policies could be also supportive with respect to developing the market for climate bonds. For example, if central banks accept green bonds as collateral, rating firms would rate climate bonds higher. Thus, indirectly via the collateral channel, central banks are able to lower capital costs and to ease credit constraints for investments in the great green transition. Central banks could ease credit flows after disasters, thereby helping to overcome bottlenecks in the supply of goods and services, while also facilitating the rebuilding of infrastructure, transport systems and other services of public interest.

While central banks can and should contribute to making the economy and the financial system more sustainable, they can only complement, not substitute for decisive political action by governments (Breitenfellner et al., 2019). However, in their role as regulators and supervisors, central banks must assess the extent to which households, firms, financial intermediaries and the stock markets are exposed to the risk of climate change and financial instability arising from devaluated prior investments in the fossil fuel sectors.

On the other hand, regulators must be aware of enormous investment opportunities resulting from the planned decarbonisation of the economy and they must contribute to easing the uncertainty around investing in the great green transition. The regulation of carbon emissions will determine the risk of devaluation. The task of financial regulators is to incorporate those risks into banking and financial markets' regulatory frameworks and to ensure financial institutions' compliance and prudential behaviour.

In addition, given the threat of 'stranded' fossil fuel assets, central banks and other supervisory bodies need to develop precautionary instruments and strategies that enable financial institutions to avoid devastating fire sales of fossil fuel assets and save the financial system a systemic crisis. Regulators should also consider a precautionary design of a public asset management vehicle, a public bad bank so to speak (Schäfer and Zimmermann, 2009), ready to digest devaluated fossil fuel assets if a system crisis emerges. In crisis times, timely reaction is key.

## Specific areas for action to foster the great green transition

### The public and private sector need to join forces

Innovation research shows that technological innovations are usually a result of a mix of private and public activities (Lamperti et al., 2019). While the public sector is required to set the pace, to a large extent, the private sector must provide the incentives, framework entrepreneurial spirit and funding. The private sources for funding green investment are numerous in principle: self-financing, corporate bond issuing, bank loans, crowdfunding and stock issuance. However, public green bonds are most likely, by far, the most important and the most effective instrument for initiating and accelerating the great green transition (Heine et al., 2019).

Most likely, a substantial part of the risk that renewable energy firms, often SMEs, face will come from incumbents unwilling to give up their profit sources. When innovators are trying to establish a new market, incumbent carbon-based oligopolies may erect and defend entry barriers against innovators in renewable energy supply. This implies that the devaluation risk for financial institutions with a progressive lending and investment policy also depends on the rules that competition policy and public authorities design and implement to keep energy markets open and contestable.

### Promotion of green technologies: Towards 100% renewable energy

With the Paris Agreement, participating countries are committed to reducing global greenhouse gas emissions by up to 90% by 2050. Beyond the nationally defined climate protection targets of its individual member states, the EU intends to promote a European energy turnaround (see, e.g. Hirschhausen et al., 2013). The Clean energy for all Europeans package (European Commission, 2019) sets the framework, aiming to promote competition for the fastest implementation and the most innovative technologies. The goal is to achieve nothing less than market leadership in the field of climate-friendly technologies. In addition to energy efficiency, emission reduction, research and innovation, Europe's objectives are also about supply security, reducing import dependency and achieving a fully integrated internal energy market.

Model results show that a transition to an energy system that relies 100% on renewables makes economic sense (Schill et al., 2018). These studies confirm that the switch to a full supply of renewable energies is not only technically feasible today but can also strengthen the economy by generating innovations and technological advantages (see Hainsch et al., 2018; Burandt et al., 2018). With more energy generated by renewable sources, costs will fall, especially for wind power and solar photovoltaics. Decreasing storage costs continue to promote competitiveness. Further cost reductions would result from a better networking of European regions. Uniform expansion targets for renewable energies and an optimised design of the EU internal market, as well as the sector coupling (i.e. shaping an integrated renewable energy system) of electricity, heat and transport would support cost reductions.

The framework conditions for investments are important for a full supply of renewables. This requires that expansion is neither capped nor impeded and that financing conditions are convenient. In addition, present subsidies for fossil energies must be consistently reduced in all countries and, above all, no new subsidies for nuclear or fossil energies must be granted. Due to their often weather-dependent fluctuations and flexibilities in supply, renewable energies must be well interlinked – also by means of intelligent technology. For this and for the use of storage facilities (Zerrahn et al., 2018), market conditions must be improved by removing existing barriers and by allowing for more flexibility.

### Better climate risk insurance schemes

Preventive actions and buffers designed to enhance the resilience against shocks are also important. Low income countries, regions and cities have a limited ability to issue climate bonds and enjoy little borrowing power. In addition to tax increases, policymakers and practitioners suggest facilitating risk pooling through self-insurance or some collective insurance schemes, grants from donors and disaster funds for contingencies. Yet, the issue of debt sustainability, as discussed above, also needs to be addressed.

A broader concept of risk pooling could aim at combining mechanisms of private or public insurance schemes with multilateral safety nets and regional catastrophic insurance schemes. While donor grants, fiscal and financial policies, risk pooling as well as insurance funds are a good foundation, others suggest that monetary policy should provide some insurance by committing to stepping in and providing liquidity for disaster-affected regions and countries with low incomes.

## Effective transition and international cooperation

The current pandemic crisis should motivate policymakers to build into the recovery plan resilience measures against the next crisis, the climate crisis.[1] The view that designing and implementing a policy mix to combat climate change is superior to focusing on a 'unicorn' measure is widely accepted. Moreover, relying only on market forces and hoping that pure cost-internalisation of environmental externalities will 'do the trick' is not enough. To induce an effective transition, as well as to moderate and mitigate the various issues of conflicting interests, the optimal policy should encompass fiscal instruments, targets and standards, public-private co-funding schemes, monetary policy, financial regulation, and disclosure practices.

Another important issue to consider is how the different measures to combat climate change can be scaled up. The best way is to avoid non-cooperative single country actions, to facilitate risk sharing and to encourage international cooperation. For example, the European and North American environmental trading systems could be linked in the future (Erdmann et al., 2019). Linking offers various benefits such as efficient carbon pricing for incentivising investment into mitigation options. Therefore, it could be one important long-term option for facilitating the great green transition globally. Another option is to undertake cooperative actions to accomplish a multilateral carbon tax. In this way, the contentious issue of a specific tax in countries that have no carbon tax in place can be avoided. Cooperative behaviour concerning large scale climate investments is another example.

Though underinvestment is the dominating risk for the great green transition, the risk of over-investment is, of course, also present; for example, into innovative firms that eventually fail to succeed in the markets for new energies. Yet, the dangers of disruptive effects of outdated fossil fuel technology and stranded assets are looming, with the potential to negatively affect banking systems and financial markets. The cost of a wait-and-see strategy rather than starting the great green transition immediately could be high, making itself visible in the higher frequency (and severity) of weather extremes, climate disasters and, subsequently, devaluated (stranded) assets.

1 For more information, see Climate Change Policies and COVID-19 Relief Are Not Mutually Exclusive, SCEPA, 12 May 2020, https://www.economicpolicyresearch.org/insights-blog/climate-change-policies-and-covid-19-relief-are-not-mutually-exclusive (26 May 2020).
